# Family Conflicts Are Bitter Splits That Hurt: A Qualitative Inquiry Toward Understanding the Impact of Family Issues in Functional Neurological Symptom Disorder

**DOI:** 10.3389/fpsyg.2021.652917

**Published:** 2021-05-24

**Authors:** Iram Zehra Bokharey, Urusa Fahim, Khola Tahir

**Affiliations:** ^1^Department of Psychiatry, Mayo Hospital, Lahore, Pakistan; ^2^Academic Dean Humanities and Social Sciences, Kinnaird College for Women, Lahore, Pakistan; ^3^Department of Psychology, University of Central Punjab, Lahore, Pakistan

**Keywords:** conversion disorder, family, functional neurological symptom disorder, interpretative phenomenological analysis, quarrels, unexpressed emotions

## Abstract

Functional Neurological Symptom Disorder (FNSD) or Conversion Disorder, is a fairly common diagnosis among mental health patients in Pakistan. Despite its prevalence there's a dearth of research on the phenomenon, particularly on the experience of FNSD. The study was conducted with the aim to ascertain the lived experiences of individuals with Functional Neurological Symptom Disorder (FNSD) around stressful situations in their families in Pakistan. For this purpose, a total sample of 10 participants (Women = 8; Men = 2) were recruited from the psychiatry department of a tertiary care hospital in Lahore, Pakistan. Semi-structured interviews were conducted and analyzed through Interpretative Phenomenological Analysis (IPA). The two main themes revealed in the analyses were quarrels and unexpressed emotions. The sub-themes of quarrels included quarrels with family members, quarrels within family, parental/marital discord, and quarrels with extended family members. The subthemes for unexpressed emotions were hurt, anger, sadness, and jealousy. In conclusion, this study revealed that in Pakistan, stressors related to family serve as significant contributing factors in the development of FNSD.

## Introduction

Functional Neurological Symptom Disorder (FNSD) or Conversion Disorder, previously known as hysteria or hysterical conversion has intrigued and baffled mental health professionals for a very long time. The new name assigned to Conversion Disorder by the American Psychiatric Association is Functional Neurological Symptom Disorder (FNSD), but the two terms can be used interchangeably (American Psychiatric Association, 2017).

FNSD is characterized by unexplained neurological symptoms including motor symptoms such as seizures and aphonia or sensory symptoms such as blindness that are unrelated to an underlying neurological or medical disorder (Espay et al., 2018). It tends to be more common in women (American Psychiatric Association, 2017). It has been proposed that excessive negative affect and anxiety can exaggerate a deficient top-down regulatory system, leading to psychogenic neurological symptoms (Perez et al., 2012; Morris et al., 2018).

FNSD was one of the most common disorders presenting in the psychiatry departments of tertiary care hospitals in Pakistan (Malik and Bokharey, 1999; Ahmad and Bokharey, 2013; Ijaz et al., 2017). There is a relatively high prevalence of unexplained neurological symptoms in South Asian cultures such as Pakistan, but the research in this area is very scanty (Bhavsar et al., 2016).

The first and perhaps the most influential explanation of FNSD was put forward by Freud nearly a century ago. He believed that unresolved childhood conflicts could lead to the development of hysterical conversion (Breuer and Freud, 1955). Later, the behavioral perspective maintained that FNSD symptoms are produced as a result of role enactments that have been reinforced by the environment (Kring et al., 2012). The sociocultural perspective of FNSD asserts that it tends to be more common in developing countries, the less educated and in rural areas. It has been observed that with an increase in psychological sophistication and technological advancements, symptoms of anxiety and depression have become acceptable ways of manifesting stress (Krendl and Pescosolido, 2020). On the other hand, in developing countries such as Pakistan, due to lack of psychological mindedness, the above-mentioned symptoms can go unnoticed while an FNSD symptom such as a pseudo-seizure, blindness etc. are likely to receive attention and serve as a cry for help (Bokharey and Rahman, 2013); due to reasons like lack of education and holding staunch beliefs that the symptoms are caused by black magic and paranormal activities (Hashmi and Malik, 2012). It further holds that FNSD is more common in collectivistic cultures such as Pakistan where direct expression of emotions is not appreciated on account of socio cultural factors such as the religious or family institutions (Georgas, 2010).

Each culture sees family's role in the development of disorders as a significant factor but the level of significance tends to vary across cultures (Georgas, 2010; Bokharey and Rahman, 2013). Similarly in FNSD the role of family is important considering its cultural significance. The conflict between not letting one's family down and own desires has been recognized as an important contributing factor to the development of various mental health issues (Kanaan and Jamroz, 2018). Therefore, it is important to recognize the interdependence between psychiatric and socio-cultural paradigms and processes (Leighton and Murphy, 2019), because any assessment of psychopathology as well as the selection of the most effective treatment must include an understanding of the patient's socio-cultural and familial context (Draguns and Tanaka-Matsumi, 2003; Shafi and Shafi, 2020).

### Theoretical Framework

Families play a vital role in Pakistan, not only during the formative years but throughout the individual's life. Most of our population dwells in extended family systems where not only one's parents but also grandparents, uncles, aunts, cousins, etc. live together in the same house. Thus, anything that happens to one member in the family impacts all other family members' social lives and psychological well-being (Rafique, 2017). The interaction between the family members and its importance is well-explained by Bowen's family systems theory (FST) (1974). The theory emphasized the role of family and suggests that families so profoundly affect their members' thoughts, feelings, and actions that it often seems as if people are living under the same “emotional skin.” (Erdem and Safi, 2018). The two suppositions on which FST works are (1) difficulty communicating with family members and that (2) people are constantly attempting to define the nature of their relationships. Using these concepts as cornerstones, FST emphasizes paying attention to sequences of interactions taking place between members of the family: who is doing what to whom, where, when, and in what way is it a problem? (Johnson and Ray, 2016).

Bowen's theory gave the idea of eight concepts that focus on the inevitable states of chronic anxiety that exist within the family and the cause of family dysfunction (Haefner, 2014). These concepts are interlocking, meaning that if there is problem in any one of the domain, it may influence the other. *Differentiation of self*; was one of the main concept of Bowen's theory which focuses on the emotional matrix, responsible for binding people together. It is a varied concept ranging from having low differentiation to highly differentiated individuals. Hard core self, are those individuals who are highly differentiated, they hold strong beliefs and are non-negotiable with others, whereas pseudo self are the individuals who are negotiable, others opinions rub on them, are emotionally less flexible and more dependent on others. *Triangles*; another important concept of the theory indicates the structural complexes and emotional processes within the relationships in triads. They are focused on the patterns of interactions within the families and the role of third individual within the triad to either bring harmony or tensions in the triad. The third concept, *nuclear family emotional system*, focuses on the emotional processes within one family. It explains that the level of differentiation of self of spouses in a nuclear family largely determines the intensity of the triangles that make up that family's emotional system. Fourth concept which is the *family projection process* is closely related to nuclear family emotional systems as it focuses on two outside influences that affect the degree of adaptation required by the triad: (1) the emotional relationship of the nuclear family to its extended family, and (2) the level of anxiety that is externally applied. Family projection is a strong influence on the development of various forms of psychiatric disorders and symptomatic behavior. The next concept is the *multigenerational transmission process* which explains the projections generations have onto the next ones in order to shape their lives. If the projections are healthy, next generations are likely to lead a healthy life, and if it is otherwise, this will lead to pathology. *Emotional cuff off* explains that people often geographically move away from their family resulting in unresolved conflicts, which can further plague the future relationships. *Sibling position*, indicates the birth order of the individual and the impact it has on their marital life and decisions they make. Lastly, the theory suggested that *societal regression* in the form of increased population leading to scarce resources, poverty, environmental issues etc. puts the pressure on the family and causes anxiety resulting in conflicts (Rabstejnek, 2008; Haefner, 2014).

### Significance of the Study

Each culture has its own significance and the people of the particular culture tend to follow its patterns inherited by them through generations. Similarly, the trend toward mental health through cultural lens holds its significance as the behavior of people tells you a lot about its importance. In more sophisticated cultures such as Western cultures there is relatively better understanding of the mental health issues than in Eastern cultures such as Asian countries (Altweck et al., 2015). Not having enough knowledge about mental disorders hinder people from seeking help from a mental health professional (Naito et al., 2020) and this automatically implies that there's dearth of knowledge related to culturally relevant etiological factors. In Pakistan, people do not have enough knowledge about mental health (Munawar et al., 2020), which makes them unaware of the mental health problems faced by them causing them to adopt non-scientific methods such as going to spiritual healers to deal with the issues (Hashmi and Malik, 2012; Choudhry et al., 2016) before seeking medical help. Even if they seek medical help the problems are usually brushed aside either by the family members or the physicians who see it merely as an “attention seeking” tactic and try to deal with the symptoms without addressing the underlying cause. Thus, understanding the stressful situations within the lives of people with FNSD would help not only mental health professionals but also the general practitioners to deal with the underlying cause of the problem more efficiently than just simply managing the symptoms.

Moreover, in Asian countries, the importance of families is well-known and it is common for people to live in joint family system. Consequently, people belonging to Eastern cultures tend to build their lives around their families. The choices they make and the lives they lead reflect their love and attachment toward their families. On account of such strong affiliation, the stressful life events which include family members (both immediate and extended) affect the individual significantly. Keeping in view the importance of family in Asian cultures not enough literature could be found explaining the role of stressful events in families as well as the stressful relationships with the family members as a major cause of FNSD. Therefore, a need was felt to design a study which would add to the existing literature of FNSD and also have strong implications for patients seeking therapy as well. Thus, in view of the empirical and theoretical evidence cited above the present study was designed to ascertain the lived experiences of individuals with Functional Neurological Symptom Disorder (FNSD) around stressful situations in their families in the socio-cultural context of Pakistan.

## Method

This study was situated in the qualitative paradigm as it seemed best suited on account of the exploratory nature of the study. Moreover, there is a dearth of research on FNSD, hence an in-depth analysis could only be ensured by qualitative inquiry to enable the authors to address the aim of this study in a comprehensive fashion.

### Interpretative Phenomenological Analysis

The particular qualitative strategy employed was Interpretative Phenomenological Analysis (IPA), which consists of a two-stage interpretation, or a double hermeneutic, in which the participants are trying to make sense of their world and the researchers try to make sense of their worldviews (Smith and Eatough, 2007). The purpose was to understand how the participants perceived the stressful events in their families leading toward the onset of their illness.

### Data Collection Method

The data were collected through in-depth interviews which provided an environment for candid conversations around significant aspects of the participants' lives (Guest et al., 2013).

### Sampling/Participants

The sample comprised ten participants (eight women and two men) chosen through purposive sampling (For demographic details, see Table 1).

**Table 1:** The demographic characteristics of the participants.

**Pseudonym**	**Age range (years)**	**Marital status**	**Education**	**Occupation**	**Average monthly income (in Pkr)**	**Stressors**
Nasir	18–23	Unmarried	Matriculation	Small enterprise business	20,000	Arguments with his mother, parental discord, feelings of hurt
Amina	18–23	Unmarried	Intermediate	Student	80,000	Fights with sister, embarrassment, parental discord
Zarina	18–23	Engaged	Matriculation	Student	50,000	Parental discord, helplessness
Hajra	18–23	Unmarried	Grade 7	Unemployed	60,000	Parental discord leading to suicidal attempts
Rabia	18–23	Unmarried	Grade 8	Unemployed	30,000	Familial stress
Zara	30–35	Unmarried	Intermediate	Unemployed	40,000	Conflicts with external family
Tahir	24–29	Engaged	Masters	Government Employee	50,000	Conflicts with external family, helplessness, feelings of hurt, aphonia
Naurin	35–40	Married	Intermediate	Unemployed	40,000	Marital conflicts, physical and emotional abuse
Maryam	24–29	Unmarried	Bachelors	Unemployed	40,000	Sadness, Feelings of insecurity, jealousy
Sara	18–23	Unmarried	Intermediate	Student	30,000	Academic pressure by family, feelings of hurt and helplessness

### Inclusion Criteria

The participants who fulfilled the diagnostic criterion given in DSM-V for FNSD (with psychological stressor) were included in the study. The minimum age was 18 years. The participants presenting with co-morbidity were excluded. Since the interviews were conducted in Urdu, the national language of Pakistan, their proficiency in Urdu was also a prerequisite. Amongst all the referrals, 14 participants were short listed to be included in the study. However, two had reservations about audio-taping and the other two withdrew because of some family issues. Therefore, the final study included a total of 10 participants.

### Ethical Considerations

The project was approved by the Departmental Doctoral Program Committee from the concerned body. Written informed consent was taken from all the participants for being a part of this research and for audio-taping the interviews. They were informed about confidentiality and their right to withdraw at any time from the study. Moreover, they were also told that in case they developed distress and needed counseling it would be provided to them free of cost at Services Hospital, Lahore. In addition, all the identifiable information regarding the participants such as age, city, etc. was masked, while pseudonyms were used to maintain the confidentiality of the participants.

### Procedure

The first author was working as a clinical psychologist at the department of psychiatry, Services Hospital, Lahore at that time. All the psychiatrists were requested to refer all patients diagnosed with FNSD (with psychological stressor) to the first author. An interview guide was developed and the interviews were conducted by the first author in a quiet and comfortable room in the mentioned department. First, a meeting was held with the participants in which they were further assessed to double check if they fulfilled the diagnostic criteria of DSM V for FNSD. Moreover, they were informed about the aim of the study and consent was taken. All their concerns about audio-taping the interviews were answered. If they agreed then the interview time was decided. In the first in depth interview, questions were asked from the interview guide, whereas the second interview was held after listening to the recording of the first interview and jotting down any queries from the first interview. The second interview was conducted within 1–3 days after the first one and served the purpose of clarifying any information gathered in the first interview and lasted a much shorter duration. For eight participants, two meetings were held, while two participants had a total of three meetings upon their request as they said they had got in touch with some important event that they wanted to share. The overall duration of the interviews per participant ranged from an hour and a half to 3 h.

An effort was made to make the interviews seem like a conversation to put the participants at ease so that a smooth flow of information could be ensured. After each interview detailed reflective notes were written around the body language, silences, crying, or chuckling etc. of each participant. Later during analyses, this proved to be very helpful as it enabled the authors to have an additional source of authentic information.

### Data Analyses and Verification

All the recorded interviews were transcribed and several passes of the transcripts were done to develop intimacy with the data. Thereafter, free textual analysis was done in which statements which seemed to highlight a theme were extracted. Later, all the subthemes clustering under the main themes were identified. The thematic layout was then shared with the other two authors. Few suggestions were given to reorganize the themes and a consensus was reached regarding the final thematic structure (Smith, 2008). Peer review, rich thick description and clarifying researchers' bias were used as the verification criteria to enhance the credibility of the data (Creswell, 2014). For peer review, the data sheets comprising list of themes extracted from the interviews was shared along with the audit trail comprising of the entire inquiry process, journaling, memoing, and the findings with three external auditors, who had no connection with the study (Elo et al., 2014; Noble and Smith, 2015). These were clinical psychologists working at local tertiary care facilities, where they had been evaluating and treating patients with FNSD in outpatient as well as inpatient departments quite frequently. One of them was a principal clinical psychologist with a clinical experience of 18 years; the second was a senior clinical psychologist with an experience of 14 years, while the third was a senior clinical psychologist with an experience of 10 years. They went through the data and agreed with the extracted themes with minor alterations. This helped us enhance the credibility of the data. Rich thick descriptions as described by Guba and Lincoln (1994) entail description of the process in sufficient detail so as to evaluate the extent of transferability of the conclusions to other settings and people. Consequently all minor details about the process and findings were described in the method section, while verbatim quotes were included in the results section (Cameron, 2011; Forero et al., 2018). We tried to make the description and analyses of the data as explicit as we could so that the analyses could seem relevant and understandable to anyone reading it. Regarding clarifying researchers' bias we have tried to avoid using value judgments to the utmost. Our descriptions of the aim of the study, its connection to prior studies and the theoretical framework as well as its social significance have been stated quite explicitly. Furthermore, we have also outlined the model of analyses that we used in detail and shared the limitations as well as implications of this study (Noble and Smith, 2015; Borowska-Beszta, 2017). Throughout this entire process, we have been fully cognizant of the assumptions and biases for conducting this research (Cohen and Crabtree, 2008). This awareness helped us in bracketing off these biases and later paved the path for comprehensive analyses (Creswell and Miller, 2000).

## Results

**Figure 1:**
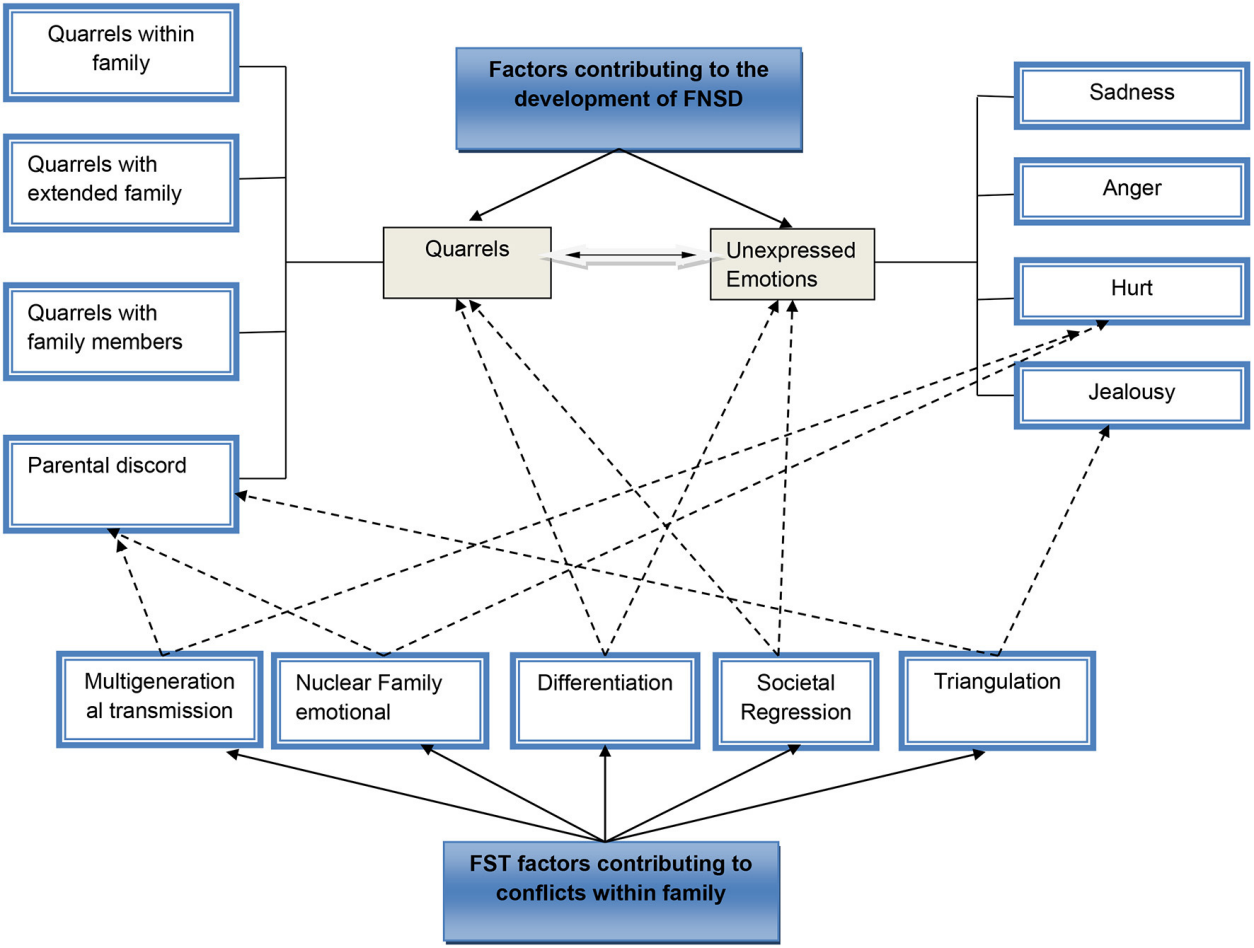
Thematic figure displaying the themes and subthemes as well as concepts of FST that contributed to the development of FNSD.

The main themes related to the connection between family dynamics and the emergence of FNSD are outlined below (see Figure 1).

### Quarrels

Quarrels and disagreements preceding the onset of symptoms was the most commonly reported theme in the interviews. At times these quarrels seemed to be an acute stressor directly linked with the onset of some participants' illness, while for others quarrels appeared to be a chronic stress factor which had been a constant pattern of maladaptive communication amongst family members. The subthemes of quarrels are described below:

#### Quarrels With Family Members

Although Nasir shared a close relationship with his mother but they often ended up quarreling with each other, which he always regretted later and caused stress for him. For instance, he said:

*I feel sad that I love my mother a lot but when I am angry I keep arguing with her endlessly… I feel remorse afterwards. After the fight, when my mother is crying, I try to amend ways with her. I feel hurt and develop symptoms*.

In Amina's case, the quarrels were with her sister. As an example of what happened when she borrowed anything from her sister she said:

*Once I wore her sandals and went outside on the road to go to my grandmother's house. She came after me and created a scene by telling me to take them off in the middle of the road and when I resisted, she started shouting at me and called me names, while people around were staring at us*.

In Nasir's case the arguments were prolonged by him and in Amina's case they were prolonged by her sister.

#### Parental/Marital Discord

Conflict between parents was another theme that was reported by most of the participants. Witnessing parents arguing and fighting, and in some cases both verbal and physical abuse, created stress among them.

For instance, Nasir said:

*When I see my parents fight, I get tears in my eyes. It has been instilled in my heart that God has given me eyes to cry and heart to feel hurt*.

Zarina reported:

*When my father hit my mother, I felt like stopping him but I felt scared and didn't understand why my father was hitting my mother as she hadn't done anything wrong? I wake up many times at night and go to check if my father had hit my mother as my grandfather used to beat my grandmother also*.

In Hajra's case the situation became so unbearable that she went to the extreme of attempting suicide on account of parental discord. According to Hajra:

…*.So, then I took an overdose. I thought let's finish the story. When my parents fight they don't realize what they are saying. I couldn't control myself and out of anger I took tablets. But the fights continued and I started having fits*.

Naurin was the only participant who was married and reported frequent quarrels with her husband as he was having an affair with a woman. Whenever Naurin confronted him he not only shouted at her but also subjected her to physical abuse. She reported:

*Once my husband came very late at night and I asked him if he had been with the other woman. He not only started shouting at me but slapped me quite hard and said to my children that I was a mad woman. The next morning when I woke up I couldn't talk*.

#### Other Quarrels Within the Family

Witnessing quarrels and discord among significant family members proved to be equally stressful as reported by several participants in this study.

Rabia's stress was related to quarrels within the immediate family which did not directly involve her but still proved to be an acute stressor leading to the onset of FNSD. Rabia's brother had frequent quarrels with their mother on the instigation of his wife mainly due to household chores:

*My brother didn't want his wife to do any household chores and wanted us to serve her. Whenever his wife complained to him about us and our mother, my brother would start fighting with mother without confirming anything, which caused a lot of stress for me*.

#### Quarrels With Extended Family

Quarrels with extended family members were also significant in creating stress for the participants of this study. For instance, Zara, in one of the statements connected a particularly stressful event to the onset of her pseudo seizures. In her own words:

*Paternal uncle said something and father got very angry. Although uncle never talks back to father but that day, he got very angry. Father told uncle to shut his mouth but he got angrier. I had never seen father and uncle so angry before. At that moment, I started trembling, fell down and had a fit*.

Likewise, Tahir's father used to get beaten up by his elder brother even when the participant was about 16. For the participant, growing up seeing father being beaten by his elder brother and not being able to do anything about it was distressing. Thus, he reported:

*My mother was very straight forward and used to provoke my father to take a stand against my uncle. I was the same height as my father, but he still got beaten up by his brother*.

### Unexpressed Emotions

Many a times, the participants had difficulty in expressing their negative emotions usually due to fear of disapproval by a family member. Ultimately these feelings bottled up and gave rise to symptoms of FNSD. These emotions are listed below:

#### Hurt

Tahir reported that his uncle (mother's brother) insulted his family publicly because of a family dispute. Other immediate family members left the room but he kept sitting there enduring the wrath of uncle as the participant didn't want to break the ties between two families. Two days after this incident, Tahir developed aphonia. According to him:

*Our maternal uncle insulted us so badly, and the younger uncle, who was also my father-in-law to be, was present there as well. All family members were crying. My mother asked us all to leave but I controlled myself and kept sitting, while my family left*.

Summing up his attitude in this unpleasant situation, Tahir said this about maternal uncle:

*He is the type of person who is addicted to cruelty. My grandfather was also like that and I think, my uncle has gotten this thing from my grandfather. And I am the type who is habituated to tolerate cruelty and never complain…*.

#### Anger

Sara's father wanted her to be a doctor while she wanted to be an artist. In order to achieve his dream, her father made her study the entire day and got angry even if Sara relaxed on festivals such as Eid (religious holiday). This really angered her but she could not express anger to her father that she did not want to study medicine. Consequently, she had first pseudo-seizure in the class. In her own words:

*I couldn't get time to relax or sleep. I felt there's nothing else in my life except studies. My cousins used to be sitting and talking, but I was not allowed to sit with them to relax and enjoy. I used to feel very angry all the time and then I had a fit in the class*.

#### Sadness

Since childhood, Maryam had been very attached to and dependent on her elder sister. When her elder sister got married, she started giving more attention to her husband, which saddened the participant. According to Maryam:

*When sister got married and went to her in-laws, she changed. However, I was expecting that she would treat me the same way as before. On the wedding reception, my sister preferred to have food with her husband which made me very sad that she has let me down in one day. If she ignored me even a little bit, I would feel sad and cry. So gradually my symptoms started*.

#### Jealousy

Hajra reported feeling jealous when father showed affection toward her brother's wife:

*After brother's wedding, my worries aggravated. My father used to show affection toward my sister in law a lot and didn't even acknowledge me. I used to wonder why he doesn't show affection toward me? So I used to get very jealous*.

Hajra's jealousy emerged in competition with her sister-in-law. She viewed it as losing the special bond that she had shared with her father.

The broad theme of quarrels represents disagreements and conflicts within families and attempts to resolve them on the part of the participants in this study. These quarrels become more significant when viewed from the lens of the Pakistani cultural context. A general sentiment found in Pakistani culture is that children must obey their parents at every stage of life and do as they are told. Thus, any conflict between parent and child is perceived as disobedience and disrespect on the child's part. Furthermore, discord among siblings and other family members, even if they are from the extended family, is discouraged. Such incidents and situations are viewed negatively from the social perspective despite their frequent occurrence.

When examined more deeply the theme of unexpressed emotions also revealed the participants' need to comply with the way they were supposed to behave. In other words, they suppressed the expression of their true feelings and emotions when they were in disagreement with the elders or with relatives who had more authority in the family. Such compliant behavior is often rewarded with praise for the individuals and for the parents. Thus, in a culture that sanctions parents to dictate every aspect of their children's lives and the lives of those who are younger in hierarchy to them, keeping one's emotions to oneself can be an act in self-protection.

In a similar manner to Braun and Clarke's (2006) suggestion, the researchers first looked at the narratives and semantics to gain an understanding of the meaning the participants were making of their experiences. Furthermore, the researchers went beyond these narratives to gain an insight into the latent assumptions behind the statements and the connections they were making (Smith and Osborn, 2015; Alase, 2017). Having an understanding of the socio-cultural context of the participants was instrumental in helping the researchers highlight the tacit patterns they noticed. These patterns were then examined to check for the researchers' biases and assumptions (Finlay and Gough, 2008).

## Discussion and Conclusion

The aim of this study was to ascertain the lived experiences of individuals with Functional Neurological Symptom Disorder (FNSD) around stressful situations in their families. We focused on the role of both immediate and extended family factors contributing toward the development of FNSD. The discussion is divided into two parts, first part comprises the findings in line with the existing empirical literature keeping in view the cultural aspects, while the second part consists of the findings discussed with reference to Bowen's family systems theory's concepts.

### Family Conflicts: The Cultural Context

The two main themes that seemed to contribute in the development of FNSD were *quarrels and unexpressed emotions. The subthemes of quarrels included quarrels with family, quarrels within family, parental/marital discord and quarrels with extended family*. This can be understood best from the lens of culture; as family has a huge importance in the life of an individual in Pakistan (Bokharey and Rahman, 2013) and generally has an influence on career decisions, choice of spouse and even selection of friends. It will not be amiss to say that one's family holds immense influence over a person's beliefs and actions. Thus, defying familial norms can lead to stress and sometimes ostracization (Mannheimer and Hill, 2014). Parents hold a very important place in the life of an individual and conflict between them can be a source of enormous stress among children and could lead to the development of various mental health issues among their children (Khattak et al., 2006; Aamir et al., 2009; Amir, 2017; Behere et al., 2017; Roizblatt et al., 2018). Moreover, it was found (Nasim, 2020) that chronically stressful situation such as parental discord is the major cause of FNSD. This is true for Pakistan where divorce is considered a taboo culturally and couples often stay in a toxic relationship due to various reasons, such as family pressure, financial issues, religion, etc. (Richa et al., 2018). Family stressors are not only limited to parental discords but also include other family dynamics. It was further indicated (Mullick et al., 2016) that communication and financial problems in family can also lead to FNSD.

Furthermore, family conflicts such as rivalry and conflicts with siblings can cause severe distress for some people (Richa et al., 2018). When the distress becomes unbearable it sometimes finds a way out in expressing their emotions in the form of physical symptoms similar to those in FNSD (Bokharey and Rahman, 2013).

The second main theme came out to be *unexpressed emotions. The subthemes comprised of hurt, anger, sadness, and jealousy*. This is in line with the previous literature which found that people who are unable to express their emotions have a tendency to develop mental health problems such as FNSD (Mullick et al., 2016; Richa et al., 2018). In addition, these unexpressed emotions are often exhibited in the form of physical symptoms, perhaps as a cry for help (Grover and Ghosh, 2014). In cultures like Pakistan, where people are less receptive toward mental health issues like depression, anxiety etc. either because of less education/ knowledge or mere negligence (Hyder, 2020), somatic symptoms experienced in FNSD tend to gather attention from the closed ones (Bokharey and Rahman, 2013). Moreover, it was indicated (Berking and Wupperman, 2012), that jealousy and possessiveness in relationships often lead to unstable mental health of the person.

We found out that the academic stress caused by the parents contributed in increasing the participant's emotional stress. These findings are well-correlated with the previous studies which have highlighted that increased academic pressure and controlled parenting are implicated in FNSD (Mullick et al., 2016; Richa et al., 2018).

### Family Conflicts: The Theoretical Context

Our findings have highlighted some concepts of Bowen' Family Systems Theory, which are imperative to the understanding of family functioning and how it impacts the stress level and mental health of individuals. As only one of our participants was married, therefore the marital discord in this case and parental discord as well as several other forms of quarrels in the cases bore similarity with Bowen's concepts. All our participants exhibited low level of *differentiation*, which was evident in their emotional reactivity to events such as attempted suicides, not getting enough attention, and not being mature enough to maintain the balance between autonomy and connection with family members; eventually led to FNSD.

In addition when spouses are less differentiated, the intensity of relationships in the nuclear family system is greater. This intense fusion or “stuck togetherness” was also evident in *quarrels*. It was found (Dallos and Vetere, 2011) that low differentiation was inversely proportional to psychological and physical health problems, which has been endorsed by our study.

The other concept that emerged as a major contributing stress factor among the participants was *triangulation*. Addition of a new family member and being ignored by a family member due to the presence of a third person caused tensions within the triad; thus giving rise to the feelings of jealousy and possessiveness. Furthermore, being involved in parental conflicts can also be extremely distressful for the individuals and can cause mental health issues. Similar findings reported (Fosco and Grych, 2008) that when conflicts within the triangles become unbearable for individuals; their mental health gets affected. Another concept of Bowen's family theory which was evident in the subtheme parental/marital conflict, was *nuclear family emotional system*. The three processes in the said concept were dysfunction of spouse (in one case), the overload of stress/anxiety, and the consequent manifestation of emotions which became evident in the form of FNSD.

The emotional system of the extended family is also a significant relationship system after the nuclear family. The interdependency of these two emotional systems is very significant. This was manifested in the subthemes hurt and parental discord. Although it was not in the scope of this study, there is some evidence of *multigenerational transmission* in the said subthemes, but it needs to be explored further. The mentioned findings are correlated with the previous literature which indicated that triangulation leads to psychological issues such as emotional distress (Fosco and Grych, 2008) which is one of the predictors of somatic symptom disorders such as FNSD in individuals, if not dealt with adequately (Oyama et al., 2007). Another concept which could partially contribute in our understanding of the development of FNSD was *societal regression*. Keeping in view the demographics of the participants, it was indicated that most of the participants belonged to lower socio economic status (SES) and had lesser education. Thus, belonging to lower SES could also be a contributing factor to the development of mental disorders such as FNSD (Social Determinants of Mental Health, 2021). The concepts of emotional cut off and sibling position didn't feature in our participants.

The findings of our study suggest that stressful situations in family are strongly implicated in the development of FNSD in Pakistan. Though the sample may have been small for generalization, it is a significant finding and needs to be replicated by further studies.

## Implications

The current study has deepened the understanding of FNSD vis-à-vis familial triggers within the cultural context of Pakistan. Gaining a deeper understanding of what triggers and sustains FNSD will be particularly helpful for the clinicians in devising effective therapeutic plans for patients with FNSD. Moreover, this study also provides a guideline in devising management strategies to identify and work on the anxiety provoking factors as explained by Bowen with an aim to heal the family relationships for long term effectiveness.

## Data Availability Statement

The original contributions presented in the study are included in the article/supplementary material, further inquiries can be directed to the corresponding author/s.

## Ethics Statement

The studies involving human participants were reviewed and approved by the Departmental Doctoral Program Committee of the University of Punjab. The patients/participants provided their written informed consent to participate in this study.

## Author Contributions

IB conceptualized the study, conducted interviews, transcribed, and analyzed them. UF was involved from the beginning and gave valuable input regarding analyses and the write up. KT helped in the literature search and the final write up. All authors contributed to the article and approved the submitted version.

## Conflict of Interest

The authors declare that the research was conducted in the absence of any commercial or financial relationships that could be construed as a potential conflict of interest.
